# Anasarca in Newly Diagnosed Type 1 Diabetes: Review of the Pathophysiology of Insulin Edema

**DOI:** 10.7759/cureus.7234

**Published:** 2020-03-10

**Authors:** Marc Wong, Tharmmambal Balakrishnan

**Affiliations:** 1 Internal Medicine, Singapore General Hospital, Singapore, SGP

**Keywords:** insulin, oedema, anasarca, edema

## Abstract

Insulin edema is a rare complication of insulin therapy that can occur after the initiation of insulin. Various timelines to the initiation of insulin have been reported after insulin therapy. Here, we report the occurrence of generalized edema in a 40-year-old woman early after the initiation of insulin. Significant differentials were excluded and resolution achieved after two weeks with diuretics. We reviewed the current literature and the possible mechanisms behind this phenomenon.

## Introduction

Insulin is the cornerstone of the management of patients with insulin deficiency in Type 1 diabetes mellitus, and its clinical use can result in adverse effects such as weight gain and hypoglycemia. Rarely, significant sodium-induced fluid retention can occur with insulin therapy, resulting in an edematous state. The mechanisms pertaining to this is less well-understood but is postulated to be related to the anabolic action of insulin that increases vascular permeability resulting in edema. Here, we report an unusual case of insulin edema resulting in anasarca in a young female after the initiation of insulin glargine and glulisine for newly diagnosed type 1 diabetes and explore the possible mechanisms behind it.

## Case presentation

A 40-year-old woman presented with a headache and was found to have a sinusitis-related headache pretreated with antibiotics. On admission, she was found to have random venous glucose of 33 mm/L and glycated hemoglobin (HbA1C) of 13.1%. (Table 1). She had a significant family history of diabetes, and her father was diagnosed to have diabetes mellitus (DM) before the age of 40. Her weight at baseline was 48 kgs, with a calculated body mass index of 18.5. Detailed history revealed that she did have mild polyuria and polydipsia with the onset of sinusitis-related symptoms. Her vitals were stable, and the initial physical examination was unremarkable. She was started on oral hypoglycaemic agents on admission but soon failed despite adequate doses and was diagnosed with type 1 diabetes mellitus. She was continued on her antibiotics for the treatment of sinusitis and commenced on insulin glargine 16 units at night, glulisine five units three times before meals, and metformin. She was discharged promptly after three days as she recovered from her sinusitis and was competent with the self-administration of insulin.

**Table 1:** Initial blood and urine investigations

Initial investigations		Reference range
Creatinine (24-hour), urine (mmol/day)	6.39	5.3 – 15.9 mmol/day
Protein (24-hour), urine (g/day)	0.15	0.0 – 0.15 g/day
Urine Protein/Creatinine Ratio	0.18	Normal < 0.20, Nephrotic > 3.0
Electrolytes		
Urea, serum (mmol/L)	4.2	2.7 – 6.9 mmol/L
Sodium, serum (mmol/L)	142	136 – 146 mmol/L
Potassium, serum (mmol/L)	4.3	3.6 – 5.0 mmol/L
Chloride, serum (mmol/L)	105	100 – 107 mmol/L
Bicarbonate, serum (mmol/L)	25.9	19 – 29 mmol/L
Glucose, serum (mmol/L)	33	3.9 – 11.0 mmol/L
Creatinine, serum (umol/L)	50	37 – 75 umol/L
Phosphate, serum	0.91	0.94 – 1.50 mmol/L
Endocrine		
Osmolality, serum (mmol/kg)	291	275 – 301 mmol/kg
Thyroxine (T4) Free, serum (pmol/L)	15.6	8.8 – 14.4 pmol/L
Thyroid Stimulating Hormone (Mu/L)	1.79	0.65 – 3.70 Mu/L
C-peptide (uG/L)	0.25	0.78 - 5.19 uG/L
Full blood count		
Haemoglobin (g/dL)	11.9	12.0 – 16.0 g/dL
WBC Count (x10^9/L)	6.22	4.0 – 10.0 x 10^9/L
Platelet Count (x10^9/L)	332	140 – 440 x 10^9/L
Lipids panel		
Cholesterol Total, serum (mmol/L)	4	< 5.2 mmol/L
Cholesterol HDL, serum (mmol/L)	0.86	> 1.0 mmol/L
Triglycerides, serum (mmol/L)	1.29	< 1.70 mmol/L
Cholesterol LDL, Calc (mmol/L)	2.55	< 2.60 mmol/L
Routine		
HBA1c, blood (%)	13.1	4.6 – 6.4%
Liver Function Test		
Protein Total, serum (g/L)	51	68 – 85 g/L
Albumin, serum (g/L)	30	40 – 51 g/L
Bilirubin Total, serum (umol/L)	4	7 – 32 umol/L
Alkaline Phosphatase, serum (u/L)	98	39 – 99 u/L
Alanine Transaminase, serum (u/L)	7	6 – 66 u/L
Aspartate Transaminase, serum (u/L)	8	12 – 42 u/L
Autoimmune		
Anti Islet Cell antibody	Negative	NA
Anti Glutamic Acid Decarboxylase antibody	Negative	NA

On Day 4, she was readmitted, as she developed generalized swelling. It started off with lower limb swelling and progressed to develop discomfort over her groin and lower abdominal area due to swelling. This swelling swiftly progressed to involve the upper limbs, abdomen, and torso the following day. She remained ambulant and did her usual activities and was compliant with her medications. She was not taking any other herbs or over-the-counter medicines when she went home. Her vitals were stable and her weight increased to 55 kgs by this time. Physical examination revealed a generalized edematous woman with marked pitting edema extending over all four limbs and face. Her heart sounds were dual and lung sounds were clear. The examination of her abdomen was unremarkable, apart from the soft tissue swelling. The examination did not reveal a shifting dullness, organomegaly, or lymphadenopathy although the patient reported an increase in her abdominal girth. She was started on gentle diuresis and with the working diagnosis as fluid overload while investigations to rule out renal complications were underway. Investigations (Table 1) revealed hypoalbuminemia on the first admission.

**Figure 1:**
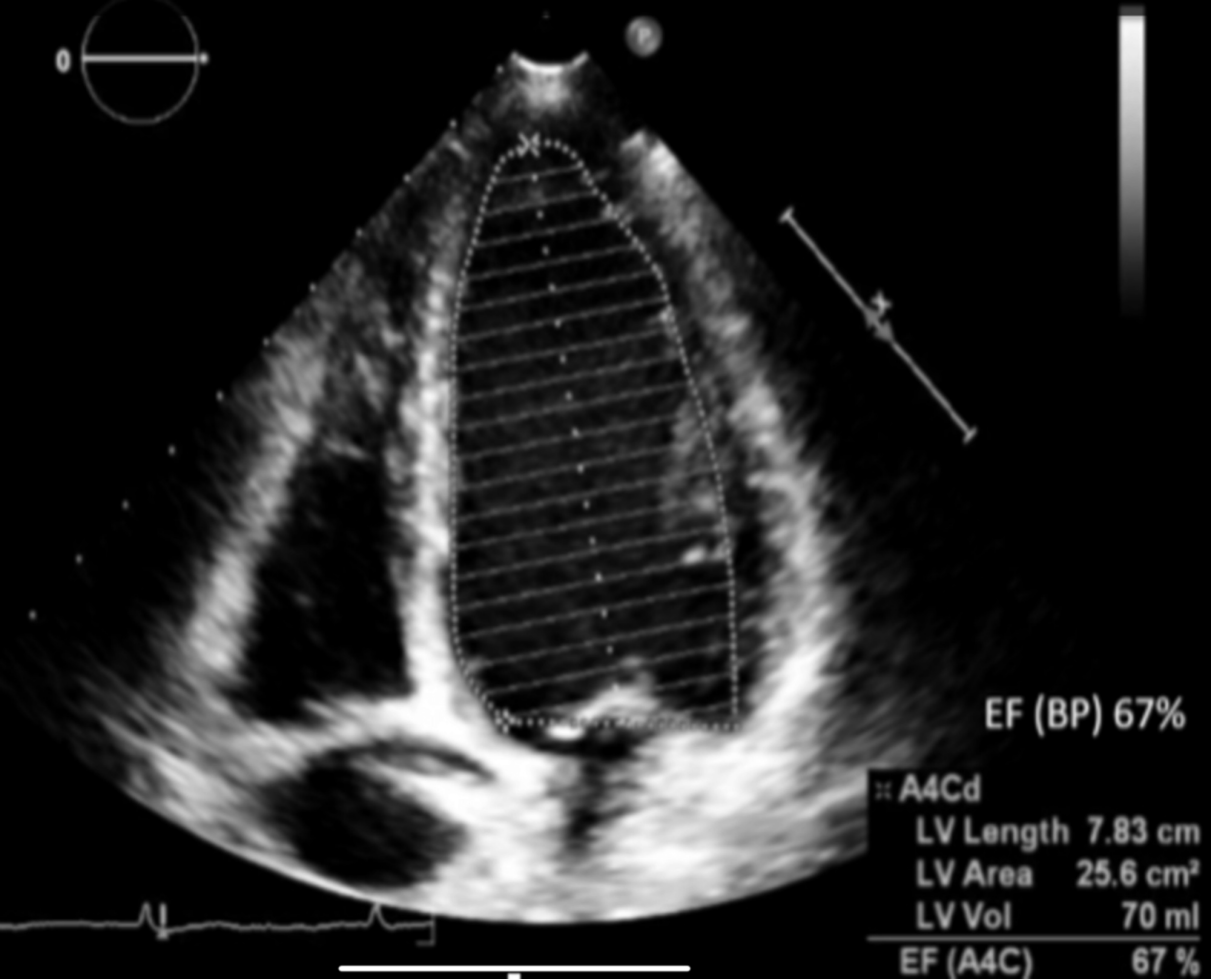
Transthoracic echocardiogram: apical 4-chamber view, LVEF of 67% as calculated by Biplane Simpson's method BP: biplane Simpson's method, A4Cd: apical four-chamber diastolic view, LV: left ventricle, EF: ejection fraction

Her renal function tests, liver transaminase, 24-hour urine protein, and thyroid function were all within acceptable ranges. Transthoracic echocardiogram demonstrated a normal left ventricular function with an ejection fraction of > 60%, without diastolic dysfunction and absent regional wall motion abnormalities (Figure 1).

**Figure 2:**
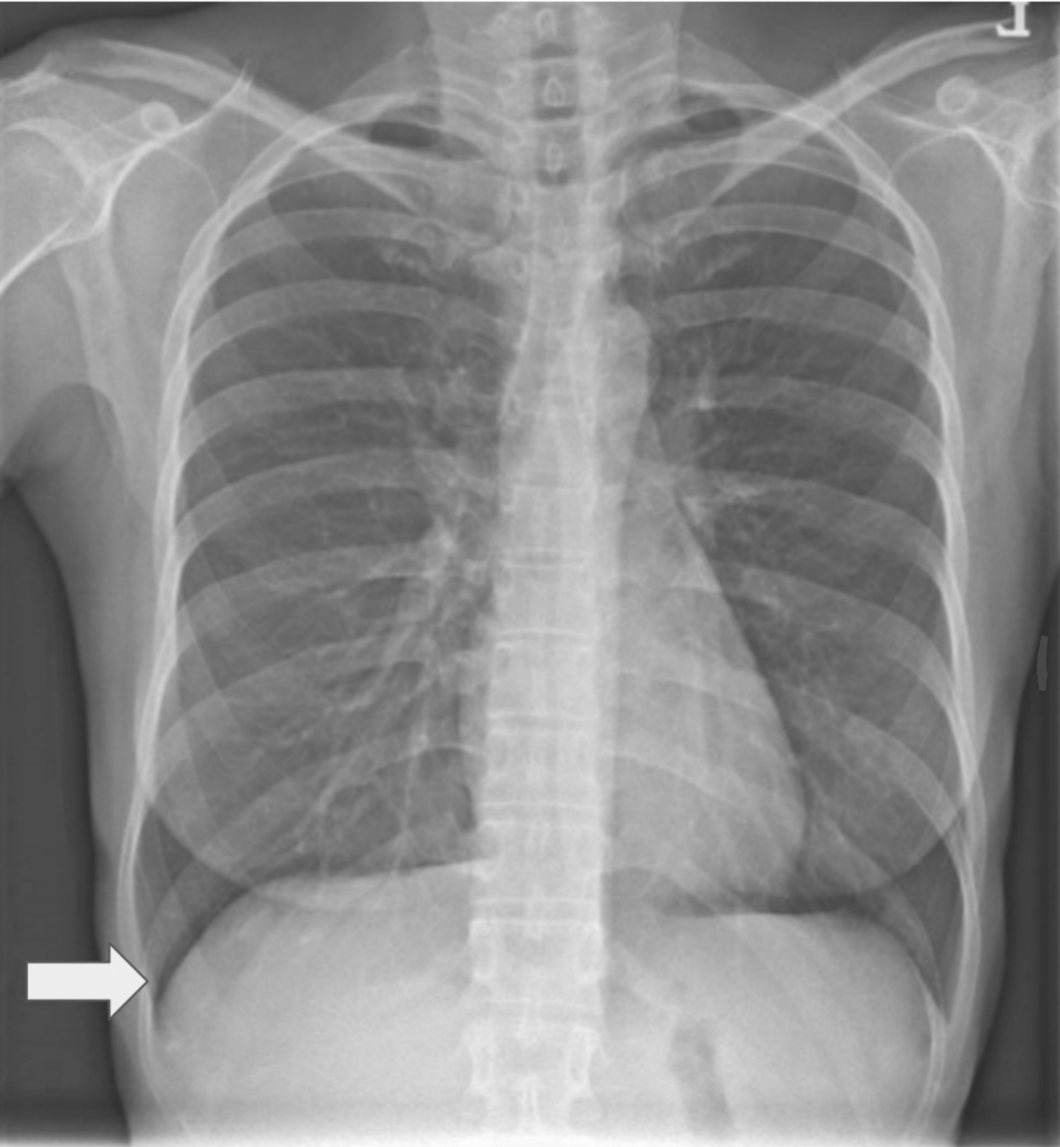
Erect chest X-ray The cardiomediastinal silhouette is normal. There is no focal lung consolidation or pleural effusion seen. The arrow points to sharp diaphragmatic borders.

Her chest X-ray showed a normal-sized heart without signs of fluid overload (Figure 2).

**Figure 3:**
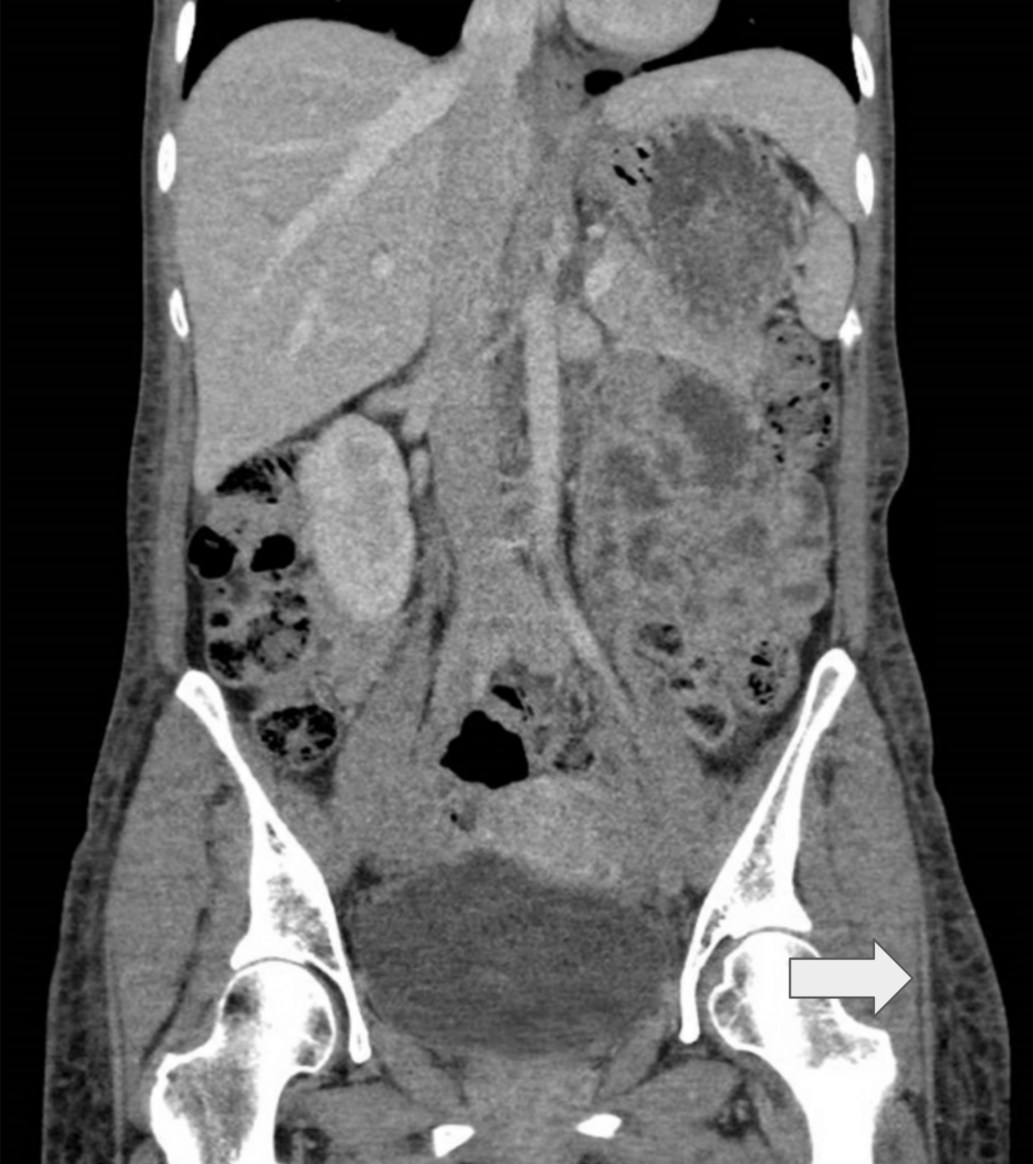
Contrasted computed tomography scan of the abdomen and pelvis A bilateral small amount of pleural effusions, diffuse subcutaneous edema, and mild interlobular septal thickening in the visualized lung bases may be related to fluid overload. The arrow points to subcutaneous edema.

A contrasted computed tomographic (CT) scan of her abdomen and pelvis was performed to rule out an obstructive cause, as the swelling started in both her lower limbs. The CT scan revealed bilateral pleural effusions with a sliver of ascites and marked diffuse subcutaneous edema (Figure 3). Notably, her liver was normal on imaging.

Other blood tests for retroviral and autoimmune causes were all unremarkable. Her weight continued to rise to a maximum of 56 kgs (8 kgs above her baseline weight of 48 kgs) despite salt and fluid restriction, and she was subsequently started on low-dose spironolactone. By now, the results of the initial investigations were largely unyielding, hence, the possibility of insulin edema by a diagnosis of exclusion was considered. This led to withholding rapid-acting insulin and starting of both dapagliflozin and spironolactone. Two days after stopping insulin, there was symptomatic relief and resolution of anasarca with her weight down-trending. She was discharged and reviewed in the outpatient clinic one week after with her weight returning to the baseline of 49 kgs and was asymptomatic. Her insulin was restarted and diuretics were discontinued after two weeks. She did not experience any recurrence of edema thereafter.

## Discussion

Insulin edema is an underreported physical finding following the initiation or intensification of insulin therapy. It was initially reported in 1928 in a 41-year-old male patient, following which subsequently reported cases were few and far in between [1]. Recently, this finding was shown to occur after the intensification of insulin, not just after initiation [2]. The true incidence of insulin edema remains unknown but some reports have estimated it to be in the range of 3%-3.5% [3-4].

The majority of reported cases of insulin edema have occurred in patients with type 1 diabetes typically presenting after starting on insulin or the intensification of insulin regimes [4-5]. There has been a recent report of such occurrence also seen in patients with type 2 diabetes on thiazolidinediones [6]. The severity of edema is usually mild, involving the limbs, sparing the torso and face. Resolution is usually seen after one to two weeks of conservative treatment with fluid and salt restriction and may or may not involve the use of diuretics [4-5]. Severe occurrences with generalized anasarca, pleural effusions, and ascites are rare but have been reported [7]. Our report is the first local representation of an Asian individual with extensive peripheral edema with recovery. Besides its relevance as a description of an unexpected treatment outcome, this case has interesting implications in the context of the debate on the mechanistic basis of insulin-induced edema.

The mechanisms resulting in insulin edema can be grouped into three categories. First, the deficiency of insulin results in a catabolic state, which potentiates the development of this edema [5,8]. The development of edema is primarily seen after the initiation of insulin replacement in newly diagnosed type 1 diabetics [4]. Chronic hyperglycemia in the catabolic state has been shown to result in increased capillary permeability through the leak of albumin into the interstitial fluid [9]. Notably, our patient had hypoalbuminemia on presentation (Table 1) and recovered with conservative management. Intensive fluid resuscitation during the initial phase of treatment may lead to extravasation of the fluid into the subcutaneous tissue, exacerbating edema [7].

A similar, rare complication of edema has also been described in patients with anorexia nervosa one to two weeks following increased food intake. This, as we now know it as refeeding edema, shares similar pathophysiology to edema seen in an insulin-deficient catabolic state [5,10]. Moreover, the severity of edema negatively correlates with BMI, with the most severe occurrence of cases of edema occurring in the severely underweight patient, further suggesting the link between the resolution of the catabolic state upon the commencement of insulin and the development of edema [7]. In our case, this was excluded both clinically and biochemistry wise.

Next, the action of insulin promotes sodium retention contributing to the edematous state. Insulin therapy promotes renal tubular sodium reabsorption by stimulating the Na+/K+-ATPase as well as the expression of Na+/H+ exchanger 3 in the proximal tubule and is balanced by glucagon, which serves to increases natriuresis at the distal tubules [11-12]. The actions of insulin when reintroduced into the insulin-deficient state leads to the promotion of sodium retention and the inhibition of natriuresis by the suppression of glucagon. This retention of sodium together with the increased capillary permeability works in tandem to promote the edematous state.

Finally, the resumption of GnRH and estrogen production contributes to further vasodilation. Chronic stress during the insulin-deficient catabolic state has been suggested to relate to gonadotrophin-releasing hormone pulse-generator dysfunction in the hypothalamus, which is responsible for the high incidence of menstrual disturbance in patients with type 1 diabetes [13]. With insulin therapy, the catabolic state normalizes, resuming the production of gonadotropins and estrogen. This may cause estrogen-induced nitric oxide synthesis in endothelial cells, leading to the rapid vasodilation of the capillary beds and the exacerbation of the third spacing of fluids [14]. 

The management of insulin edema requires the early identification of patients at high risk for the development of severe complications such as serosal effusions and cardiac failure [7]. A female patient who is underweight is at the highest risk as are other patients with poor cardiac, hepatic, or renal reserves [3]. For these patients, fluid and salt restriction should be implemented early, which may be all that is required. Diuretic therapy can be used in more severe decompensated cases. From the pathophysiological standpoint, an aldosterone antagonist, such as spironolactone, may be preferred considering the presence of inappropriate hyperaldosteronism, although therapy with other diuretics appears to be as effective [4-8,15]. The reintroduction of insulin necessary for the management of type 1 diabetes should be gradual and accompanied by a frequent reassessment of fluid status. In another report, the initiation of a type 1 diabetic on an insulin pump for the intensification of therapy was met with insulin edema and treatment-induced neuropathy [16]. However, the exact timeline for the resumption of insulin, however, remains unknown. Some have considered the use of ephedrine, currently only reported in one case; this would require further evidence to support its clinical use in this setting [17]. 

In our patient, the mild edematous reaction was extensively evaluated and finally attributed to insulin edema, a diagnosis of exclusion. The transient hypoalbuminemia seen was consistent with the leakage of albumin into the interstitial fluid. There was no reason to stop insulin-like in our case, as this will disrupt the glycemic control. Insulin edema, a benign complication of insulin therapy that is under-recognized and under-reported. Better recognition, understanding, and early management of this condition will provide some relief from anxiety in affected patients, most of whom are young, with newly diagnosed diabetics. This can contribute to enhanced compliance with insulin therapy essential for the long-term management of type 1 diabetes.

## Conclusions

Insulin edema is a rare complication of insulin therapy that can occur after the initiation or intensification of insulin predominantly in the patient population consistent with underweight young females. Significant differentials of the refeeding syndrome and malnutrition should be considered since they have similar pathophysiology. With appropriate short-term diuretics and salt restriction, this condition can be treated, with a good prognosis. The recurrence of insulin edema has not yet been reported. This study leaves us with some take-home messages: (1) generalized edema is a possible side effect after the initiation or intensification of insulin regimes; (2) insulin edema is a diagnosis of exclusion; (3) underweight female patients with poor cardiac, renal, or hepatic reserves are at high risk; (4) salt restriction can be advised as a measure to prevent and treat insulin-induced edema; and (5) insulin-induced edema responds well to diuretic therapy.
